# Multi-Scale Sheep and Goat Face Detection Network Based on Multi-Task Optimization

**DOI:** 10.3390/ani16172685

**Published:** 2026-08-27

**Authors:** Fu Zhang, Baoping Yan, Xiaopeng Zhao, Sanling Fu, Yakun Zhang, Tianhua Chen

**Affiliations:** 1 College of Agricultural Equipment Engineering, Henan University of Science and Technology, Luoyang 471023, China; 230410260090@stu.haust.edu.cn (B.Y.); 250320261664@stu.haust.edu.cn (X.Z.); zhangyk@haust.edu.cn (Y.Z.); 2 State Key Laboratory of Light Superalloys, Henan University of Science and Technology, Luoyang 471023, China; 3 College of Physical Engineering, Henan University of Science and Technology, Luoyang 471003, China; 4 College of Biological and Agricultural Engineering, Jilin University, Changchun 130025, China; chenth22@mails.jlu.edu.cn

**Keywords:** sheep and goat, face detection, RetinaFace, lightweight, feature fusion

## Abstract

Face detection serves as the prerequisite and foundation for individual sheep and goat identification. Considering the practical detection process, the facial region of sheep and goats shares similarities with the torso texture features, which could be easily interfered with by the background and other factors, along with the presence of varying face scales. RetinaFace was selected as the framework, with GhostNet used as the backbone for feature extraction, thereby significantly reducing model complexity and computational load. A new feature extraction unit—SRM module—was designed through the integration of the Multi-Source Domain Adaptation (MSDA) attention mechanism and Reparameterized Non-local Cross-scale Feature Exchange Layer 4 (RepNCSPELAN4) to mitigate the impact of environmental factors and enhance the fine-grained feature extraction of the sheep and goat’s face. A new feature fusion network—A_EFPN—was designed through the com-bination of Efficient Reparameterized Generalized-FPN (RepGFPN) and Alterable Kernel Convolution (AKConv), thereby enhancing feature interaction and improving the model’s multi-scale detection ability. An optimized multi-task joint loss function was employed to retain three facial key points: the left eye, right eye, and nose, thereby reducing the number of model regression parameters. Experimental results demonstrate that the proposed method achieved 96.15% Precision, 97.06% Recall, 96.60% F1 Score, 98.31% AP, 2.40% NME, 2.36 G FLOPs, 3.63 M Params, and a weight of 14.75 MB. It was demonstrated to be applicable to the rapid and accurate detection of sheep and goat faces in the actual breeding environment, providing the foundation for subsequent sheep and goat face recognition.

## Simple Summary

Individual sheep and goat identification is essential for precise feeding and intelligent management, which not only reduces labor costs but also improves the precision of farm management. A lightweight sheep face detection method based on the RetinaFace framework is proposed in this paper, which simultaneously performs bounding box detection and key point localization of sheep and goat faces, making it suitable for practical detection environments and providing a foundation for subsequent sheep and goat face alignment and recognition. The results will serve as an important support for personalized feeding plans, health analysis, and smart farm construction.

## 1. Introduction

The modern sheep and goat farming industry is rapidly advancing towards large-scale and intensive development. Sheep and goats are raised in high-density environments, and improper management can lead to a series of issues such as environmental degradation and the outbreak of widespread diseases, which presents new challenges and demands for feeding and management methods [1]. Individual detection of sheep and goats is key for tracking and tracing them, which not only reduces labor costs but also improves the precision of farm management. It also serves as an essential support for individualized feeding plans, health analysis, and construction of smart farms [2]. Therefore, individual sheep and goat detection is crucial for monitoring and precision management.

Traditional animal identification is based on methods such as branding, ear tagging, and embedded microchips. Although traditional methods offer simplicity and efficiency, they may inflict harm on animals, and the markers can become increasingly difficult to identify over time [3,4]. With the wide application of deep learning algorithms in the field of target detection and other areas, the use of image target detection technology to detect faces can realize the rapid identification of animal identity in complex environments [5,6]. References on pigs and cattle are shown in Table 1.

In terms of sheep and goat face detection, Xue et al. [12] proposed a SheepFaceNet sheep face recognition network based on Euclidean spatial metrics to solve the problems of more redundant information in sheep face images and poor poses and angles of sheep faces, which enabled the detection and alignment of sheep face regions in the images. Billah et al. [13] produced two different datasets for detecting the faces of sheep with high similarity, eyes, nose and ears, with accuracies of 93%, 83%, 92% and 85%, respectively. Zhang et al. [14] proposed a sheep face recognition model with an efficient channel attention mechanism that incorporates spatial information with an accuracy of 97.91%. Ning et al. [15] introduced the SimAM parameter-free attention mechanism and the CARAFE up-sampling module in YOLOv5s, which was able to accurately recognize individual dairy goats with similar facial features. Zhang et al. [16] proposed a sheep face recognition model based on the Vision Transformer (ViT) architecture, called ViT-Sheep, which introduced the LayerScale module and used transfer learning, achieving a sheep face recognition accuracy of 97.9%.

Most of the current sheep and goat face detection algorithms transform the recognition task into a classification task. Specifically, the facial images of each individual of the same kind are viewed as a class, and then a deep convolutional neural network is used to realize the classification task [17,18]. In this process, it is necessary to use a large number of images to pretrain a classifier, and then input the target to be recognized into the classifier for classification, and ensure that the training set contains all the images of the target to be recognized, and at the same time, the classification labels correspond one-to-one with the category of the species. Since the classifier has thousands of dimensions, this causes it to be unable to recognize individuals outside of the training set, and therefore it is very limited in its ability to be trained again to recognize new individuals when they appear. At present, face recognition technology based on deep learning has matured and is widely used in various fields such as transportation, security, healthcare, and education [19,20,21,22]. With reference to the human face recognition process, it is typically divided into three phases: first detection, then alignment, and finally recognition, with clear task division, higher accuracy, and better applicability. Sheep and goat face detection serves as the premise and foundation of sheep and goat face recognition. Due to the presence of multi-angle and multi-scale sheep and goat faces, as well as the similarity of sheep and goat texture features in actual farming environments, significant challenges are posed to detection and localization performance for sheep and goat faces. Therefore, a lightweight sheep and goat face detection method based on the RetinaFace framework is proposed in this paper, which simultaneously performs bounding box detection and key point localization of sheep and goat faces, making it suitable for practical detection environments and providing a foundation for subsequent sheep and goat face alignment and recognition.

## 2. Related Work

### 2.1. Dataset

**Figure 1:**
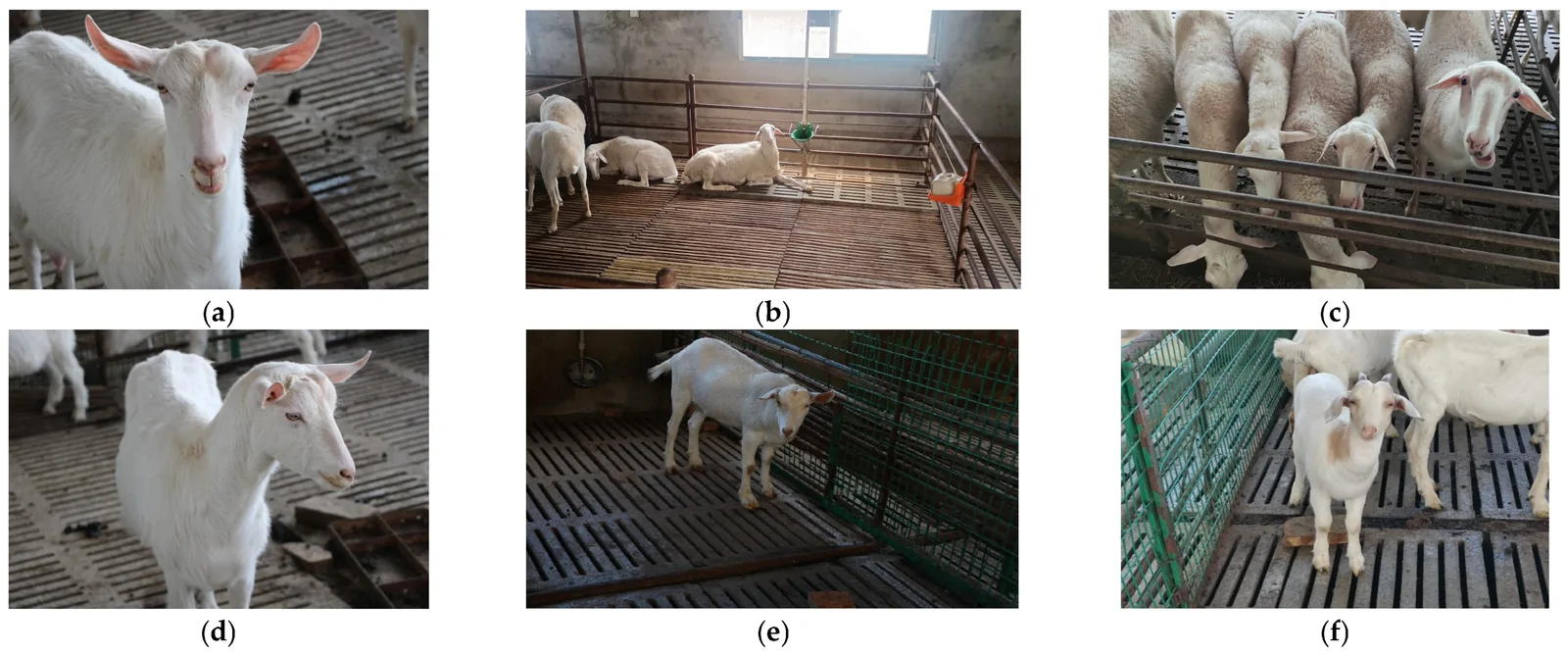
Sample of sheep and goat face image. (**a**) Positive face, (**b**) remote, (**c**) occlusion, (**d**) side face, (**e**) dark light, and (**f**) lambs.

The sheep and goat images used originated from Xiangshun Agricultural and Animal Husbandry Science and Technology Company Limited, Luoning, Luoyang, China. The Canon (Canon EOS 750D, Canon Inc., Suzhou, China) DSLR camera with the frame rate set to 30 f/s and a cell phone were used to supplement the photography to obtain images of sheep and goats in their natural state in an actual farming environment. In order to simulate the complex environment of a real sheep and goat farm, the collected data include images of different angles, different distances, different light intensities, occlusions, lambs, single sheep and goats, and flocks, ensuring data diversity. Key frames were extracted from the original video data at intervals of 25 frames using FFmpeg, and images containing sheep and goat faces were retained. A portion of the data is shown in Figure 1.

### 2.2. Data Preprocessing

**Figure 2:**
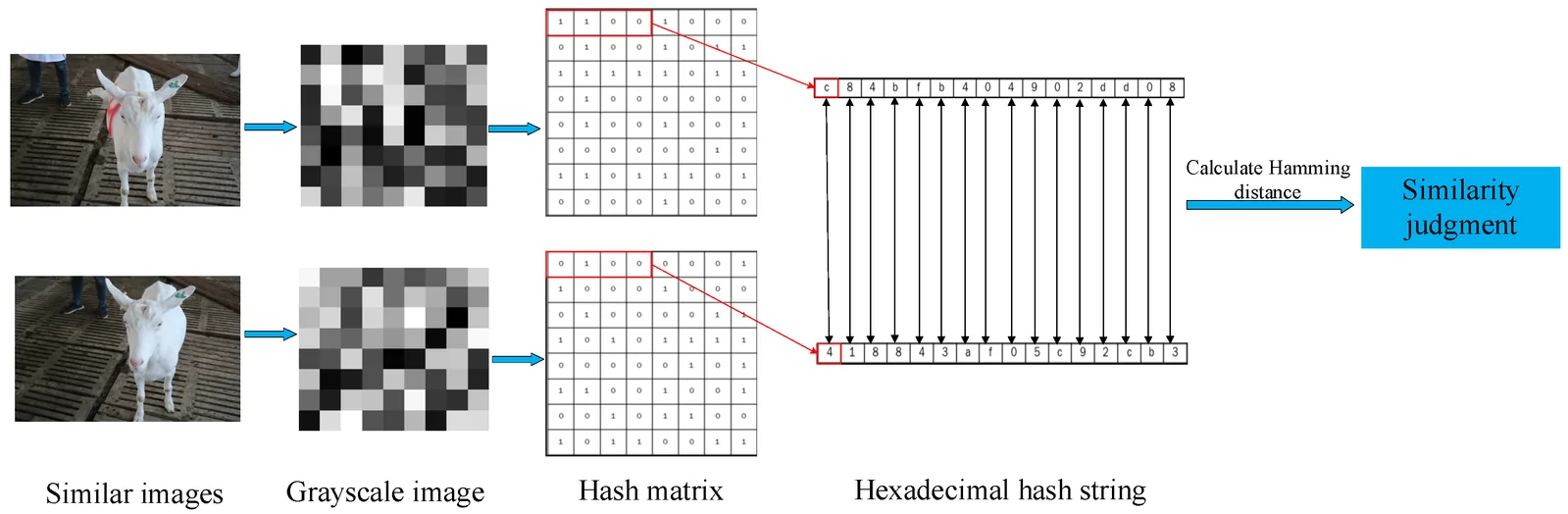
Flowchart of the hash algorithm.

To address the issue of excessive similarity between consecutive frame images, a difference hash algorithm (d-Hash) was employed to calculate the hash values of two images. The Hamming distance between these hash values was then compared to assess their similarity. Images with high similarity were subsequently removed. The specific process is illustrated in Figure 2. Initially, the input images were resized to 9 × 8 pixels to reduce the data size. Subsequently, the resized images were converted to grayscale to minimize the influence of color information and focus on variations in image brightness. Next, each row of pixels was traversed to compute the brightness differences between adjacent pixels. If the brightness of the previous pixel was greater than that of the subsequent pixel, the difference value was set to “1”. Otherwise, it was set to “0”, resulting in two 8 × 8 binary hash matrices. Finally, both hash matrices were converted into hexadecimal strings to represent the hash values of each image. The Hamming distance between these hash values was then calculated, with smaller distances indicating greater similarity between the images. In this study, a Hamming distance threshold of 10 was set. If the Hamming distance was less than 10, one of the images was deleted. If it was greater than or equal to 10, both images were retained. Additionally, images with blurred sheep and goat faces, or significant loss of sheep and goat face information, were excluded from the remaining dataset. After screening, a total of 2906 original images were obtained. According to the statistics of the number of sheep and goat faces, there were 1947 single-face images, 587 double-face images, and 372 multi-face images.

### 2.3. Data Augmentation and Annotation

#### 2.3.1. Data Augmentation

**Figure 3:**
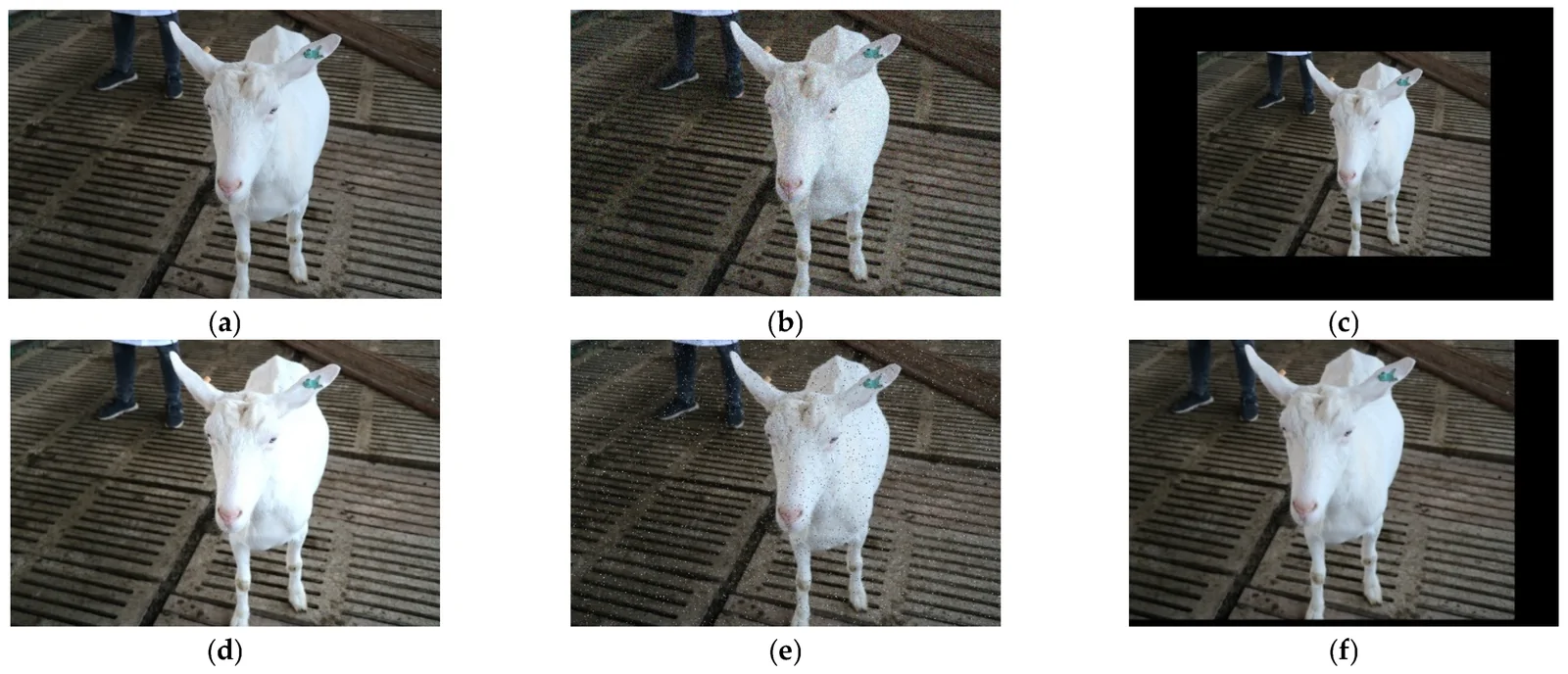
Partial image enhancement effect. (**a**) Original image, (**b**) gaussian noise, (**c**) random scaling, (**d**) brightness adjustment, (**e**) changing pixel points, and (**f**) translation.

Training neural network models requires a substantial amount of data, as insufficient data volume may result in important features being overlooked, overfitting, and other issues. To enhance the generalization ability of the deep learning model, the original samples were augmented through data enhancement to improve data diversity and richness. A total of 2906 original images were subjected to Gaussian noise, random scaling, brightness adjustment, pixel point change, translation, and other operations. Each original image generated 5 enhanced images, combined with the original images to form a total of 17,436 sheep and goat face detection images. Based on 2906 original images, the training sets and the test sets were divided according to an 8:2 ratio. The training set contains 2325 original images and their enhanced images, a total of 13,950 images, and the test set contains 581 original images and their enhanced images, a total of 3486 images. Some of the enhanced images are presented in Figure 3.

#### 2.3.2. Data Annotation

**Figure 4:**
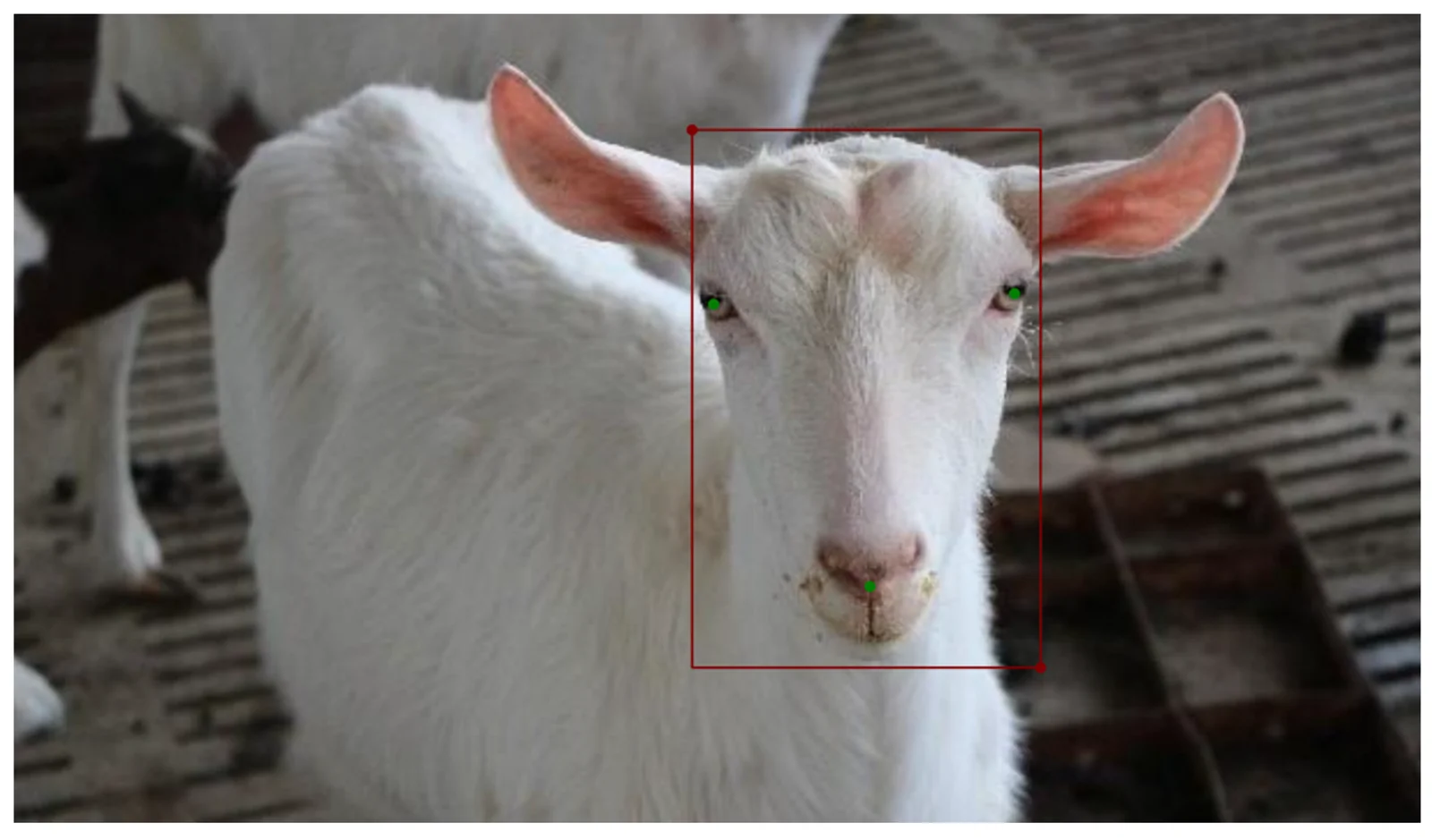
Data labeling process.

Labelme (5.4.0) software was used to annotate the sheep and goat face images. The top-left corner coordinates were connected to the bottom-right corner coordinates to create a bounding box around the sheep and goat’s facial region, ensuring that the entire sheep and goat face appeared within the target box, while minimizing the target box size to reduce the impact of cluttered backgrounds. In the human face detection process, five facial key points, namely the left eye, right eye, nose, left mouth corner, and right mouth corner, were labeled. Observations of sheep and goat face images revealed that the primary facial features were concentrated within the area defined by the two eyes and the nose. Additionally, in frontal views of the sheep and goat, the left and right mouth corners were frequently obscured and poorly defined. Therefore, the left eye, right eye, and nose were chosen as the key points for annotation, effectively localizing the sheep and goat’s facial region and preparing for subsequent sheep and goat face alignment. The annotation process is shown in Figure 4.

After annotation with Labelme software, the corresponding JSON file for each image was generated, containing the coordinates of the upper-left and lower-right corners of the sheep and goat face frame, as well as the coordinates of the three facial key points: the left eye, the right eye, and the nose. Since the label file for model training follows the Wider Face detection dataset format, a Python (3.12) program was developed to read the information from the JSON file, which was converted into the Wider Face dataset format and stored in a TXT file containing the image path and sheep and goat face location information.

## 3. Sheep and Goat Face Detection Network

### 3.1. RetinaFace Network

**Figure 5:**
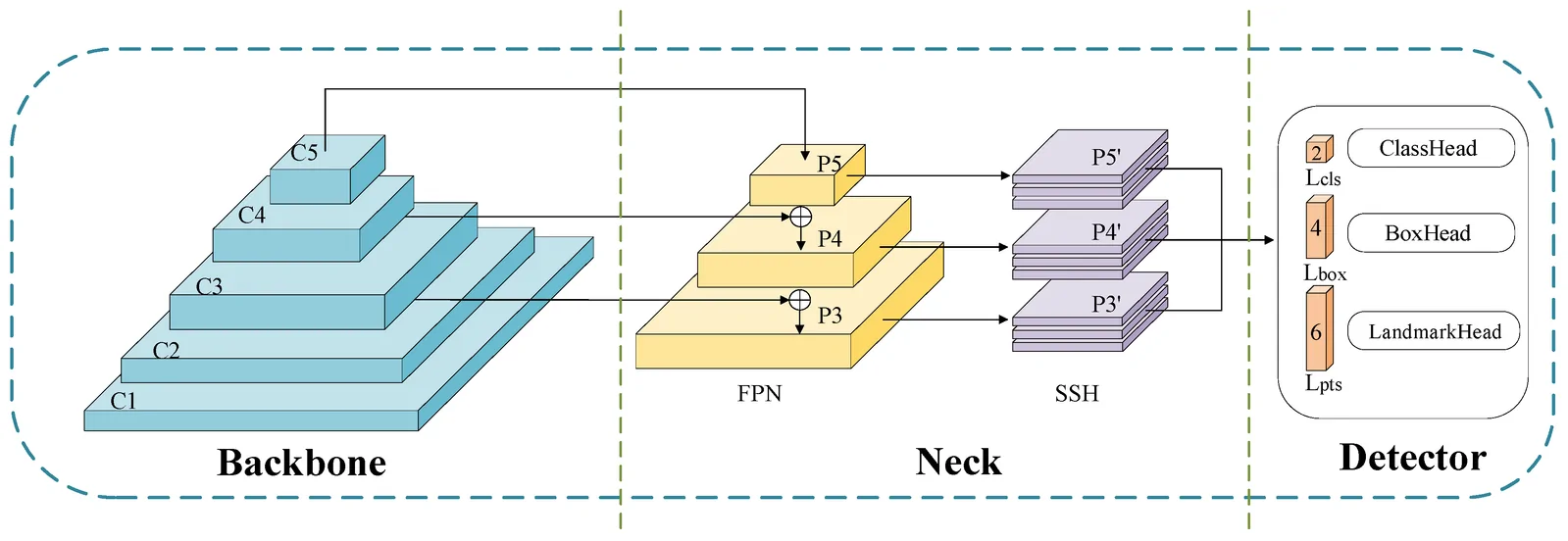
RetinaFace network structure diagram.

To achieve efficient and accurate sheep and goat face detection in the complex environment of a sheep and goat farm, the RetinaFace [23] network model was adopted as the foundational framework for sheep and goat face detection in this study, referencing methods used in human face detection. RetinaFace is a single-stage face detection algorithm, consisting of three parts: the backbone network, the feature fusion module, and the predictor, as shown in Figure 5. The original RetinaFace employed ResNet50 as the backbone network. The feature fusion module was composed of a feature pyramid network (FPN) and a single stage headless (SSH) context module. The predictor included sheep and goat face classification, sheep and goat face bounding box regression, and sheep and goat face key point regression.

The target image was first passed through the ResNet50 backbone network to extract multi-scale features and obtain the effective feature layers C3, C4, and C5. These feature layers were then input into the feature pyramid network through lateral connections to fuse feature information from different layers. Higher-level features were combined with lower-level features through a top-down path to obtain rich semantic information. The three effective feature layers P3, P4, and P5 were obtained through the feature pyramid network, and then input into the context module to enhance the receptive field and generate the final feature layers P3′, P4′, and P5′. Finally, sheep and goat face classification, bounding box regression, and key point regression were performed through the detection head.

### 3.2. Improved RetinaFace Network

**Figure 6:**
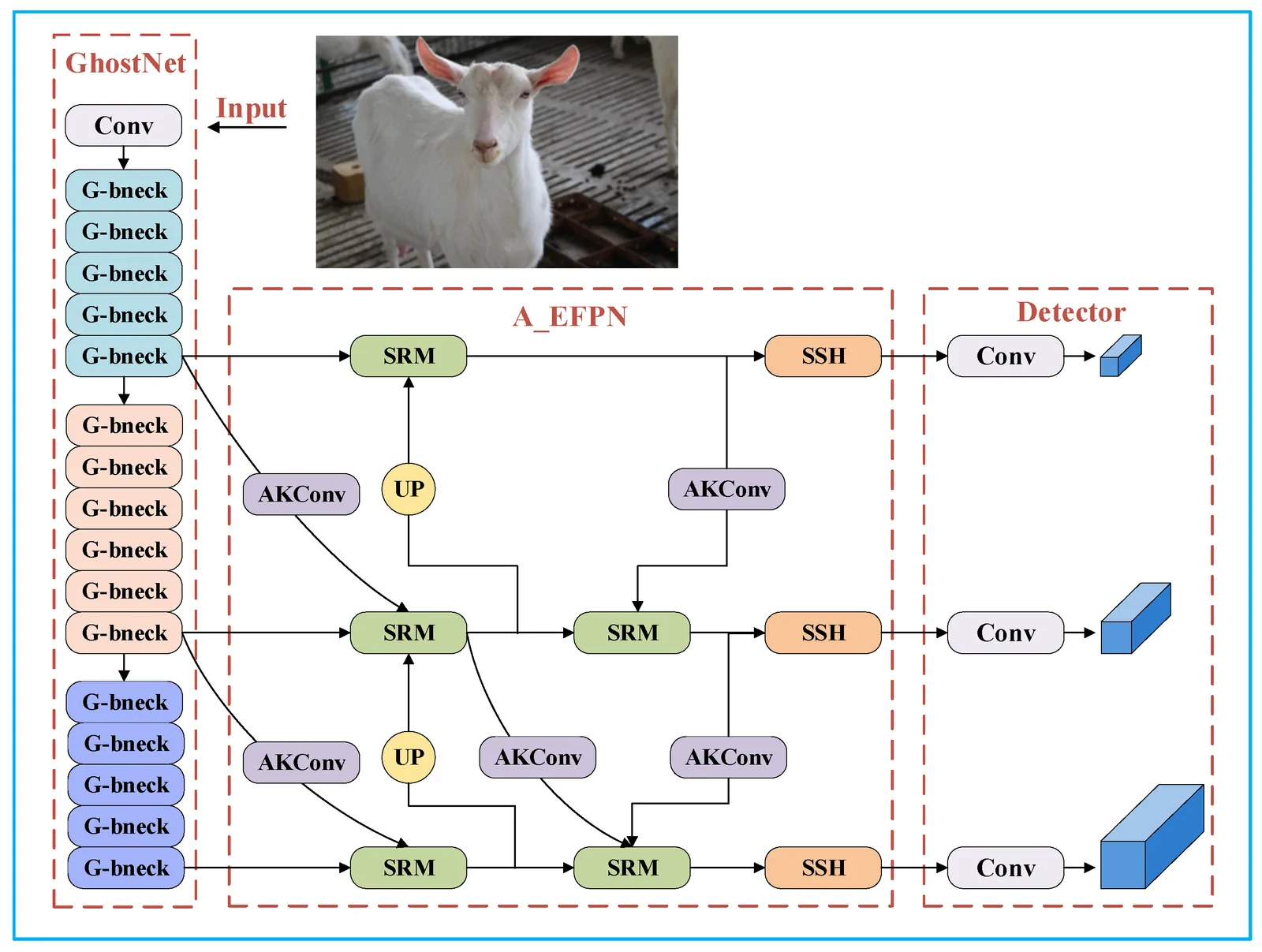
Improved RetinaFace network structure.

To reduce model complexity and computational load, GhostNet was employed as the backbone network for feature extraction, enabling rapid detection of sheep and goat faces. To address the issues arising from uneven lighting distribution in actual sheep and goat farm environments, where the distinction between the sheep and goat’s facial area and the body was unclear, and texture features were similar, the MSDA attention mechanism [24] was integrated with RepNCSPELAN4 to design the SRM module. This module was a key component of the feature fusion module, mitigated environmental influences, and enhanced the extraction of fine-grained features related to sheep and goat faces. To address the excessive number of sheep and goats in large-scale breeding environments, the presence of varying sheep and goat face sizes results in leakage and false detections. By combining Efficient RepGFPN and AKConv [25], the A_EFPN feature fusion network was designed, replacing FPN, to improve the model’s multi-scale detection ability. In order to adapt to the sheep and goat’s facial area characteristics, the multi-task joint loss function was optimized, while retaining the left eye, the right eye, and the nose as the three facial key points to reduce the number of model regression parameters. The improved RetinaFace network structure is shown in Figure 6.

#### 3.2.1. GhostNet Backbone Network

**Figure 7:**
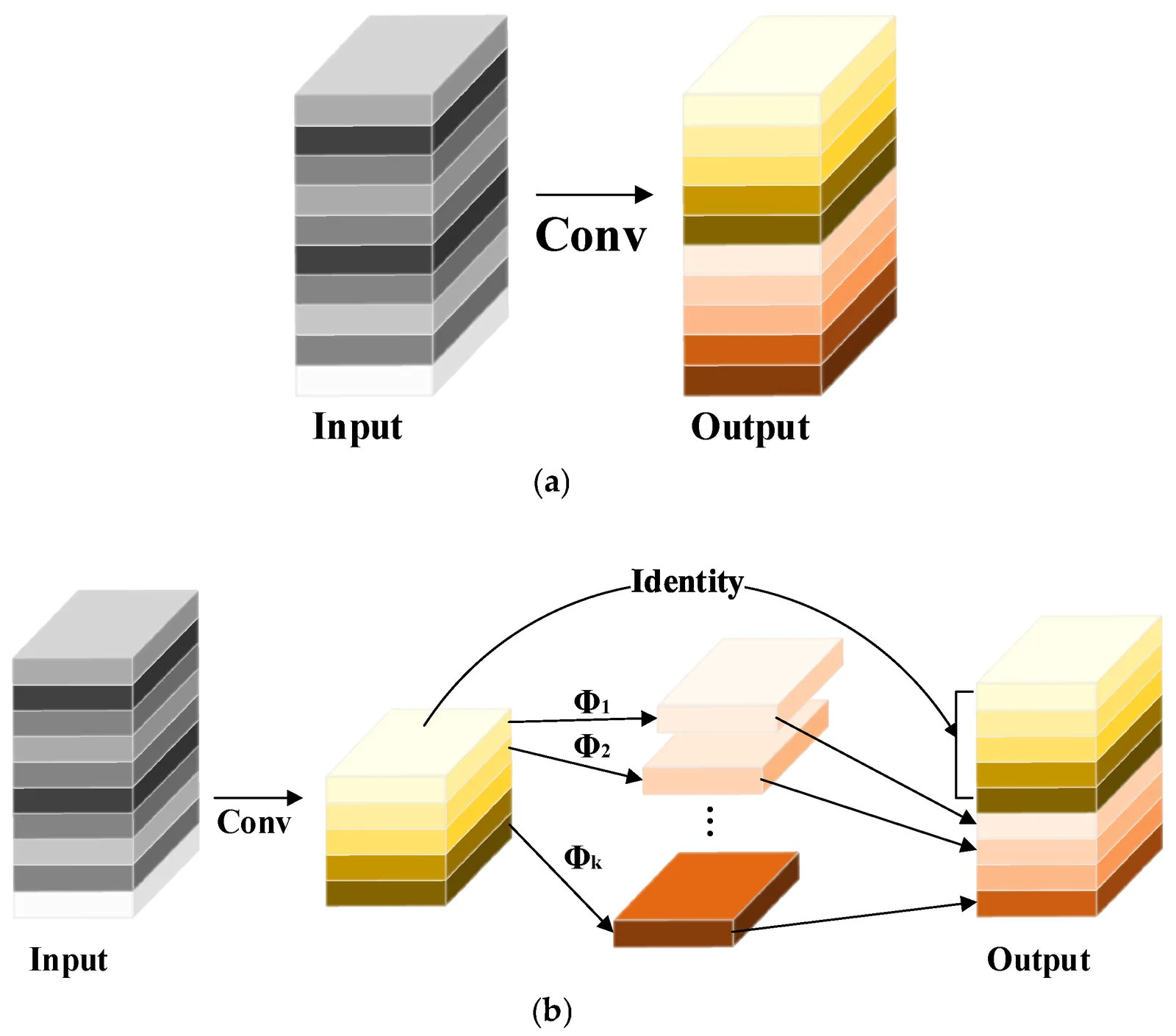
Conventional convolution with Ghost module. (**a**) The convolutional layer. (**b**) The Ghost module.

GhostNet is a lightweight network architecture designed to address the issue of feature redundancy in neural networks by introducing a Ghost module that generated “ghost features”. The core idea was to first generate basic features through fewer convolutional operations and then generate more features through a series of simple linear transformations, thus reducing the computational complexity of the network. The ordinary convolution and Ghost modules are shown in Figure 7a,b.

If the Ghost module is used to generate the same number of feature maps as a conventional convolution, given the input feature map size of $h^{\prime }\times w^{\prime }\times c$ and the output feature map size of $h^{\prime }\times w^{\prime }\times c$, with a convolution kernel size of k×k, the average kernel size for linear operations as d×d, and the feature map channel compression factor as s, the *FLOPs* for conventional convolution can be calculated as follows. The computational effort with the Ghost module is reduced to approximately 1/s times that of a normal convolution. The Ghost module can be expressed as follows:

(1) $$FLOPs\left(a\right)=n\times {c\times h}^{\prime }\times w^{\prime }\times k\times k$$

(2) $$FLOPs\left(b\right)=\frac{n}{s}\times {c\times h}^{\prime }\times w^{\prime }\times k\times k+\frac{\left(s-1\right)n}{s}\times h^{\prime }\times w^{\prime }\times d\times d$$

where k×k, d×d and s were all much smaller than c. The ratio of the two was:

(3) $$r=\frac{FLOPs\left(a\right)}{FLOPs\left(b\right)}=\frac{c\times k\times k}{\frac{1}{s}\times c\times k\times k+\frac{s-1}{s}\times d\times d}\approx \frac{s\times c}{s+c-1}\approx s$$

**Figure 8:**
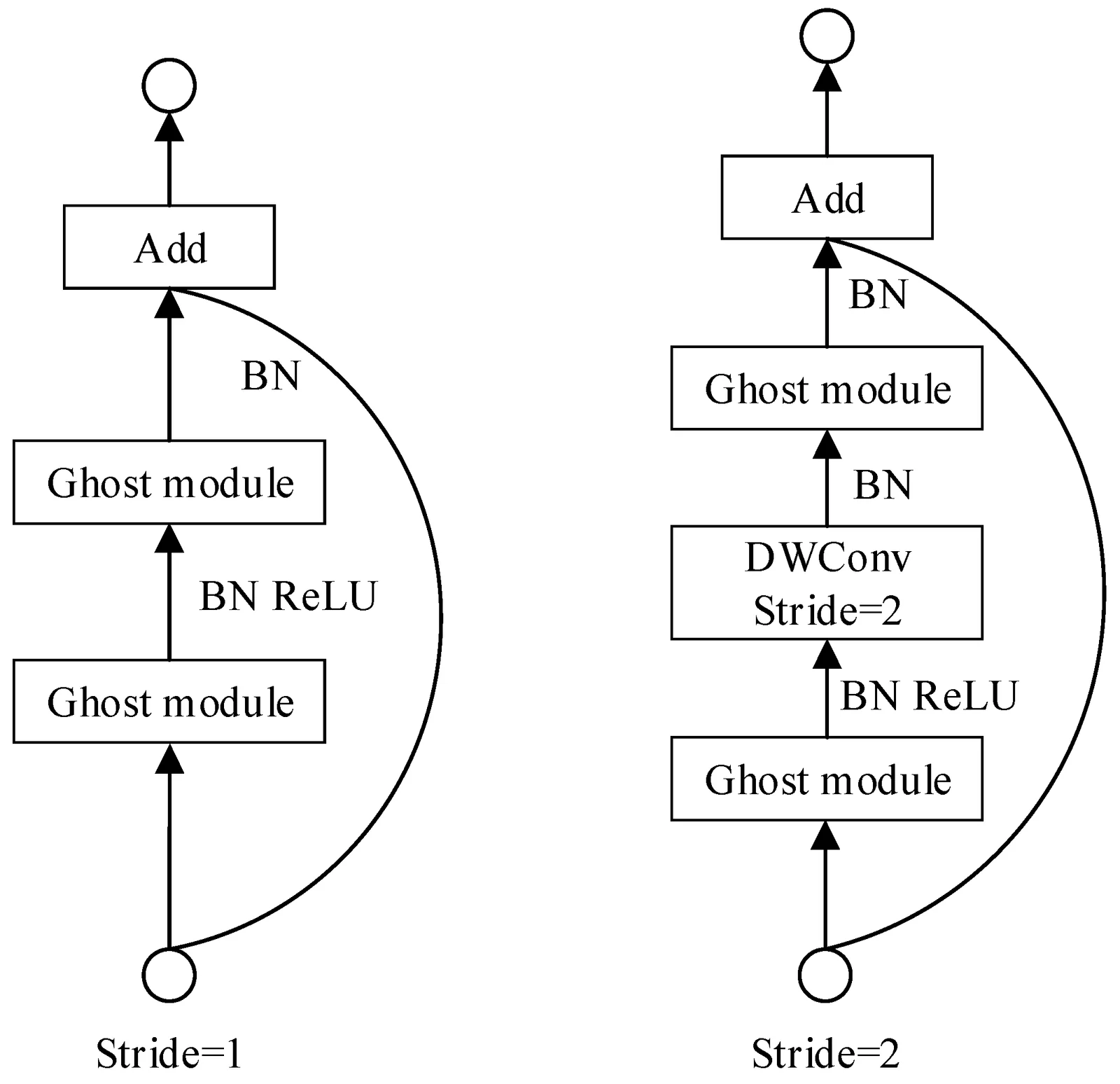
Structure of the Ghost Bottleneck.

GhostNet consists of a series of Ghost Bottleneck (G-bneck) stacks, and the network parameters are shown in Table 2. To enhance the multi-scale detection ability of the model, the input image size was set to 640 × 640 × 3, and the feature maps of 80 × 80 × 64, 40 × 40 × 128, and 20 × 20 × 256 were taken as the output of the backbone network. The Ghost Bottleneck mainly consists of Ghost modules, which include two types: one with a stride of 1 and the other with a stride of 2, as shown in Figure 8. The input feature map undergoes feature extraction and channel modification through the Ghost module, while a shortcut branch directly connects the input to the output of the Ghost module. This design reduces the computational load of the model and minimizes the loss of feature information during inter-layer transmission.

#### 3.2.2. SRM Feature Extraction Module

**Figure 9:**
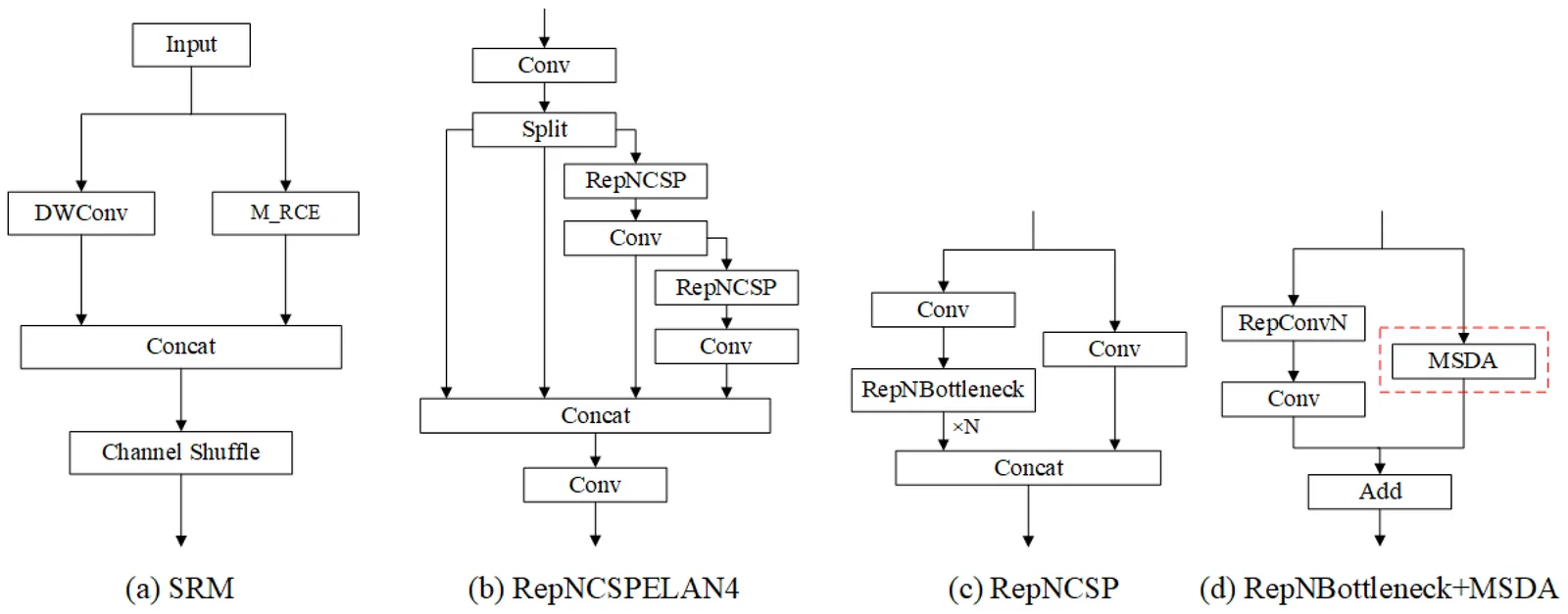
SRM structure.

To address the issue that the actual detection process was susceptible to background interference and other factors, as well as the similarity in the color of the sheep and goat’s facial region and the torso, which led to inaccurate positioning of the sheep and goat face bounding box and key points, RepNCSPELAN4 was integrated. Additionally, the MSDA attention mechanism was introduced in the residual linkage, and the SRM module was designed by incorporating the Channel Shuffle technique, which reduced the environmental influence and enhanced the extraction of fine-grained features of the sheep and goat’s face. The SRM module is shown in Figure 9a, and M_RCE denotes the RepNCSPELAN4 module of the fusion attention mechanism.

The input feature map was passed through the 1 × 1 Depthwise Separable Convolution (DWConv) to perform linear combinations of different channels, enhancing information interaction among the channels. At the same time, the M_RCE module strengthened feature extraction, improving the representation of spatial and contextual features to obtain fine-grained characteristics. Through a concatenation operation, shallow spatial information was combined with deep semantic information to acquire global features of the target. Finally, the Channel Shuffle operation randomly shuffled features along the channel dimension, breaking channel isolation, thereby avoiding redundancy and interdependencies between feature channels, which enhanced the model’s generalization capability.

##### Feature Extraction Unit

RepNCSPELAN4 mainly consisted of RepNCSP and RepNBottleneck, as shown in Figure 9b–d. By combining the ELAN idea with a multi-branching structure, it could capture multi-dimensional feature information under different sensory fields, and the branching structure allowed the features to pass effectively through the network, which enhanced the feature representation ability of the model. The network depth was increased by stacking multiple sub-modules to enable it to capture deeper features, thus improving the recognition of targets in complex scenes. The reparameterization technique was introduced to use multiple different convolutional branches during the training phase and merge the multi-branch convolutional kernels into one equivalent convolutional kernel during the inference phase, which achieved the same feature extraction effect with less computation and improved the inference efficiency.

##### Attention Mechanism Module

**Figure 10:**
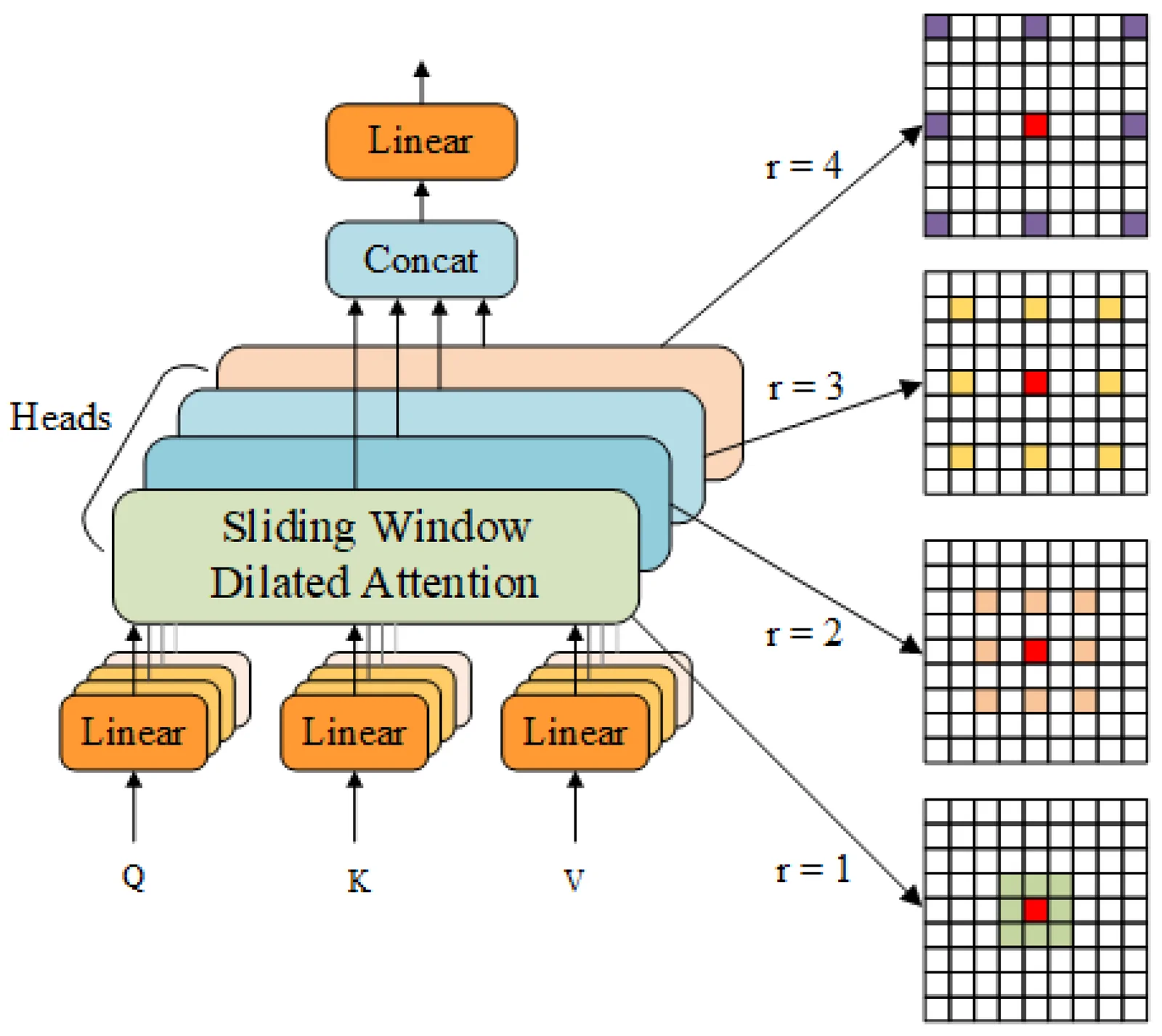
MSDA structure.

To reduce the influence of background factors and enhance the extraction of key and multi-scale features of sheep and goat faces, the MSDA attention mechanism was incorporated into the residual connections of the RepNBottleneck, as shown in Figure 9d. The MSDA structure is shown in Figure 10. Given the input feature map, the corresponding Q (queries), K (keys), and V (values) were obtained through linear projection. The channels of the feature map were divided into four different heads, with different expansion rates (r = 1, r = 2, r = 3, and r = 4) set for each head. Sliding Window Dilated Attention (SWDA) was performed at different heads with different dilation rates, and the outputs of each head were merged through the Concat operation. Finally, the merged outputs were input to Linear for feature aggregation, integrating the information learned from each head and obtaining a rich feature representation.

Formally, the SWDA operation for each head is expressed as:

(4) X=SWDA(Q, K, V, r)

where Q, K, V represents the query, key, and value matrices, and r is the dilation rate controlling the sparsity of the attention. Specifically, for each query located at position (i, j), the corresponding component $x_{ij}$ of the output X is calculated as:

(5) $$x_{ij}=\mathrm{Attention}\left(q_{ij},K_{r},V_{r}\right)=\mathrm{Softmax}\left(\frac{q_{ij}K_{r}^{T}}{\sqrt{d_{k}}}\right)V_{r}$$

where $q_{ij}$ is the query at position (i, j), $K_{r}$ and $V_{r}$ are the selected keys and values from the feature map, and $d_{k}$ is the dimensionality of the keys.

The feature map is split into n heads, and each head was assigned a different dilation rate, enabling the model to capture multi-scale semantic dependencies. The self-attention operation was performed within each head, as follows:

(6) $$h_{i}=\mathrm{SWDA}\left(Q_{i},K_{i},V_{i},r_{i}\right),1\leq i\leq n$$

where $Q_{i}$, $K_{i}$, $V_{i}$ represent slices of the feature map fed into the *i*-th head, and $r_{i}$ represents the dilation rate for the *i*-th head.

The outputs from all heads were then concatenated:

(7) $$X=\mathrm{Linear}\left(\mathrm{Concat}\left[h_{1},h_{2},\ldots ,h_{n}\right]\right)$$

This concatenated feature map X was then passed through a linear layer to aggregate the information learned from each head, generating a rich feature representation that captured dependencies at multiple scales.

MSDA utilizes the sparsity of the self-attention mechanism at different scales and focuses on features at different scales by setting different dilation rates at different heads to obtain rich contextual information and improve the model’s multi-scale detection capability. Meanwhile, the channels of the feature map are separated into multiple heads, and each head processes a different subset of features in parallel to avoid ignoring the key features of the sheep and goat face, which improves the accuracy of the sheep and goat face frame and key point localization.

#### 3.2.3. A_EFPN Feature Fusion Network

**Figure 11:**
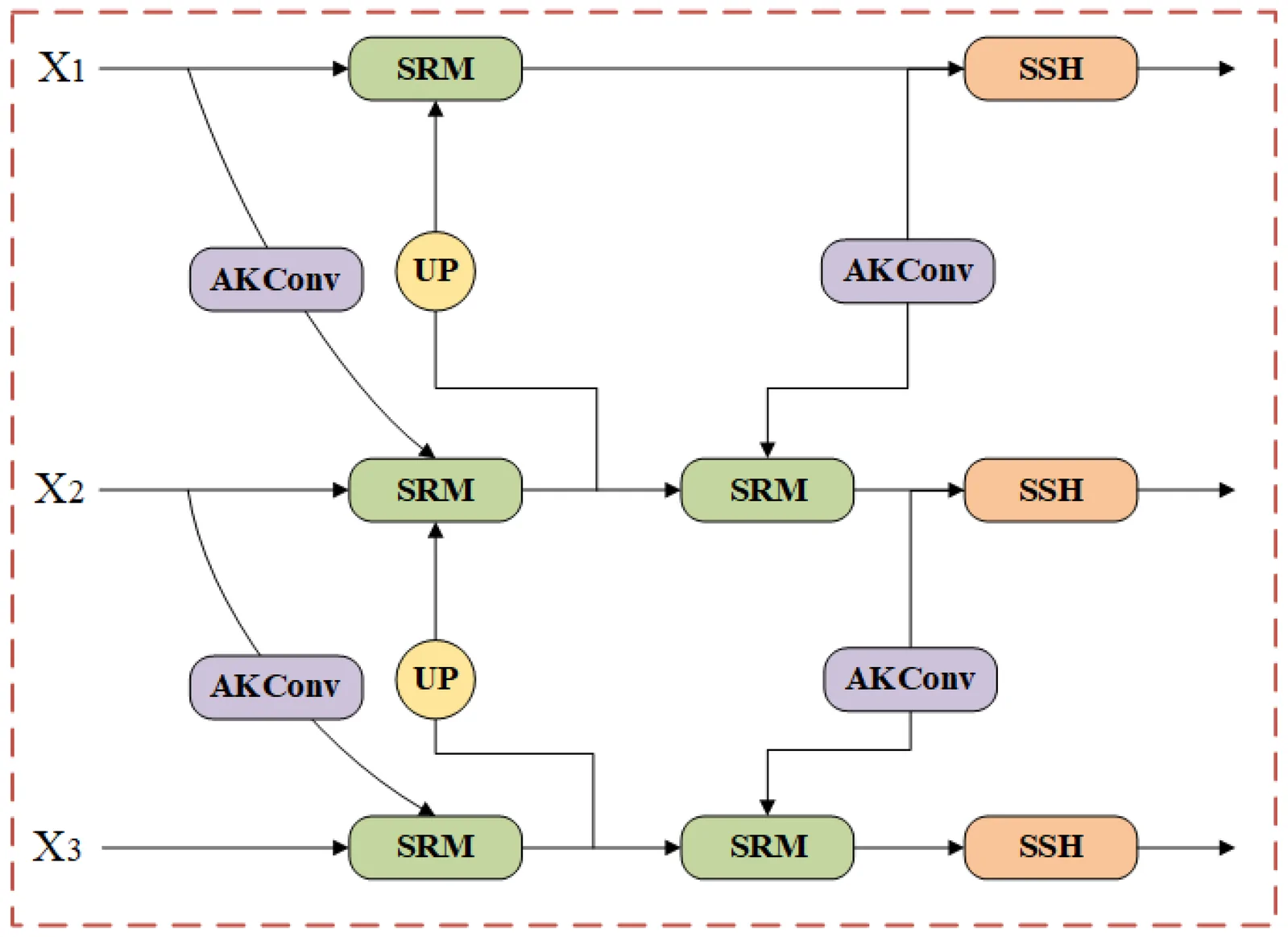
A_EFPN structure.

To enhance the model’s ability to detect sheep and goat faces of varying sizes, the A_EFPN feature fusion network was developed to substitute FPN by incorporating AKConv into the connection paths of Efficient RepGFPN, as shown in Figure 11. Due to the large depth differences among features of varying scales, direct fusion was not beneficial for representing multi-scale features. For instance, small objects were detected with large-scale features, but their shallow feature depth failed to capture detailed target information, impairing the detection of small objects. Unlike FPN, which directly fuses the three feature layers C3, C4, and C5, A_EFPN incorporated five nodes, each composed of SRM modules, to concurrently process high-level semantic information and low-level spatial information with equal priority. These nodes accepted sequential feature inputs from varying scales, both above and below, while maintaining consistent feature depth. This design greatly increased the amount of information available for feature fusion, promoting multi-scale information within consistent depths and enhancing feature interactions across varying scales and depths.

The core idea of AKConv is variable kernel convolution, which breaks through the limitations of traditional convolution confined to fixed local windows and fixed sampling shapes, and allows the convolution kernel to have an arbitrary number of parameters and sampling shapes, which enables the convolution operation to be accurately adapted to different datasets and targets. For different sizes of the convolution kernel, AKConv first defines its initial sampling shape, and through the learned offset, adjusts the initial sampling shape.

The offsets are learned by the network and represent the adjustments needed to align the kernel with the object’s shape or target area. These offsets are learned through the convolution process, similar to how deformable convolutions functioned, but with the added flexibility of irregular shapes. The convolution operation itself is used to predict the offsets at each sampling position. The offsets were not predefined but were learned dynamically by the network. The prediction process for offsets could be visualized as follows: A convolutional layer in the network generated an output that was a map of offsets. These offsets modified the positions of the initial sampling grid, causing the kernel to sample from different regions of the input data based on the learned shifts.

**Figure 12:**
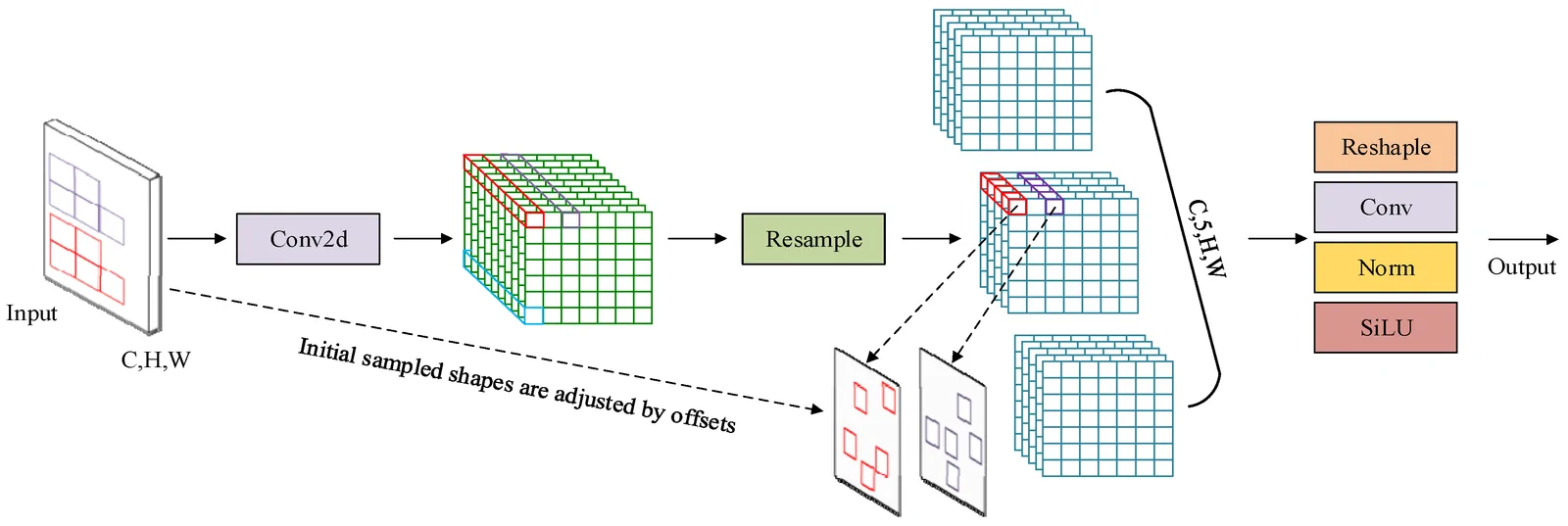
AKConv structure.

According to the adjusted sampling shape of the feature map, resampling was performed, which effectively adapted to the changes in the sizes of the targets, thereby improving the model’s multi-scale detection ability. At the same time, AKConv adopted an asymmetric convolution kernel, which supported the linear adjustment of the number of convolution parameters, contributing to model lightweighting. The structure of AKConv is shown in Figure 12.

#### 3.2.4. Multi-Task Joint Loss Function

The loss function of RetinaFace is composed of a face classification loss, a face bounding box loss, and a loss for five facial key points. According to the actual situation of the sheep and goat faces, it was found that the distinguishable features of the sheep and goat faces are mainly concentrated within the area surrounded by both eyes and the nose, and the left and right corners of the mouth are easily blocked. Therefore, the loss function for the facial key points was optimized to retain only three facial key points, namely, the left eye, the right eye, and the nose. The multi-task joint loss function is shown in Equation (8).

(8) $$L=L_{cls}(p_{i},p_{i}^{*})+\lambda_{1}L_{box}(t_{i},t_{i}^{*})+\lambda_{2}L_{pts}(l_{i},l_{i}^{*})$$

In the equation, $L_{cls}(p_{i},p_{i}^{*})$ represents the face classification loss function, which uses the cross-entropy loss to determine whether the current candidate box contains a sheep and goat face. Here, $p_{i}$ denotes the predicted label, and $p_{i}^{*}$ represents the true label, with a value of 1 indicating the presence of a sheep and goat face and 0 indicating its absence. $L_{box}(t_{i},t_{i}^{*})$ is the bounding box loss function that employes Smooth L1 to regress the position of the sheep and goat face bounding box, measuring the error between the predicted bounding box $t_{i}$ and the true bounding box $t_{i}^{*}$. $L_{pts}(l_{i},l_{i}^{*})$ denotes the key point regression loss function used for the localization of sheep and goat face features, which also utilized Smooth L1 to assess the difference between the predicted key point $l_{i}$ and the true key point $l_{i}^{*}$. $\lambda_{1}$ and $\lambda_{2}$ are the weights for the bounding box loss and keypoint loss functions, respectively. To ensure a balanced loss across tasks, both $\lambda_{1}$ and $\lambda_{2}$ were set to 0.5.

### 3.3. Model Training

In this study, a sheep and goat face detection network was built based on Pytorch framework, and Intel (R) Xeon (R) Silver 4210R CPU @ 2.40 GHz and NVIDIA Quadro RTX 5000 GPU were used for model training. Python 3.8 was used for development, PyTorch 2.1.0 was employed as the deep learning framework, and GPU-accelerated computations were performed with CUDA 12.1. The input image was set to 640 × 640, epoch was 150 rounds, batch size was 16, SGD optimizer was selected to optimize the model parameters, with momentum of 0.937 and weight decay of 0.0005. The cosine annealing learning rate descent method was used to adaptively adjust the current learning rate to a maximum of 0.01 and a minimum of 0.0001.

### 3.4. Evaluation Metrics

In order to verify the comprehensive performance of the model, Precision, Recall, F1 Score, AP, NME, FLOPs, Params and Weight were selected as evaluation indices. Specifically, the AP was computed by matching predicted bounding boxes with ground-truth annotations using the IoU threshold of 0.50. For the sheep and goat face detection task, the raw predictions were first filtered by the confidence threshold of 0.50, followed by non-maximum suppression (NMS) with the IoU threshold of 0.45 to remove redundant detections. The NME was normalized by the diagonal length of the sheep and goat face bounding box to ensure consistency in error evaluation across sheep and goat faces of varying scales. In multi-face scenarios, all valid sheep faces in each image were included in the evaluation. For detection results, a one-to-one matching strategy was adopted to determine true positives (TP), false positives (FP), and false negatives (FN). The predicted box was considered a successful detection if its IoU with a ground-truth box exceeded the predefined threshold; otherwise, it was treated as a false positive or a false negative. Based on the statistical results aggregated over all test images, Precision, Recall, and F1 Score were subsequently calculated.

(9) $$Precision=\frac{TP}{TP+FP}$$

(10) $$Recall=\frac{TP}{TP+FN}$$

(11) $$F1=2\times \frac{Precision\times Recall}{Precision+Recall}$$

(12) $$AP=\int_{0}^{1}P\left(r\right)dr$$

(13) $$\begin{array}{c} NME=\frac{1}{M}\sum_{i=1}^{M}\frac{{\left\|{\hat{p}}_{i}-P_{i}\right\|}_{2}}{d} \end{array}$$

TP refers to the number of samples correctly identified as positive, *FP* refers to the number of samples incorrectly identified as positive, and *FN* refers to the number of truly positive samples incorrectly identified as negative. Precision is the proportion of samples predicted to be positive that were truly positive, while Recall is the proportion of all truly positive samples that were correctly predicted as positive by the model. The *F*1 Score is the harmonic mean of Precision and Recall, which provide a unified assessment of both metrics. *AP* is the area enclosed by the P-R curve, which is plotted with Recall on the x-axis and Precision on the y-axis. *NME* is a metric for evaluating the performance of key point detection, calculated as the error between the predicted key point and the true key point.

## 4. Experiments

### 4.1. Comparison of Different Backbone Networks

To achieve model lightweighting, ResNet50 was replaced with MobileNetv1, MobileNetv3, and GhostNet as the backbone networks for comparative experiments with RetinaFace. To ensure the fairness of the results, all models were retrained on the sheep and goat face dataset created in this study. The results are shown in Table 3. As can be seen from the table, after replacing the original RetinaFace’s ResNet50 backbone network with a lightweight network, a certain amount of detection accuracy was lost due to the shallow depth of the model reducing the feature extraction capability, but the FLOPs, Params, and Weight were greatly reduced, which was more suitable for mobile deployments in real farming environments. In comparison among MobileNetv1, MobileNetv3, and GhostNet, although MobileNetv1 had the smallest FLOPs, Params, and Weight, it exhibited insufficient detection accuracy and a larger key point error. Conversely, GhostNet achieved the highest Precision, Recall, F1 Score, and AP, along with the smallest key point error (NME), while also meeting the requirements for lightweighting. Therefore, GhostNet was selected as the backbone network for further improvements in this study.

### 4.2. Ablation Experiments

In order to verify the effectiveness of the proposed method in this paper, the performance of the model before and after the improvement was compared and analyzed; the results are shown in Table 4. 

Compared with the original RetinaFace, after the backbone was replaced with GhostNet, the detection accuracy of the model decreased, but the model complexity was substantially reduced. With the introduction of the AKConv deformable kernel convolution, the AP was improved by 1.37% to 93.75%, and the NME was reduced to 3.21%, indicating that AKConv effectively enhanced the detection capability for geometrically deformed targets through adaptive sampling shapes. After the FPN was replaced with the A_EFPN feature fusion network, the AP was significantly increased by 3.58% to 97.33%, and the NME dropped to 2.64%, demonstrating that the feature interaction and multi-scale information fusion capability were strengthened, and the detection accuracy was recovered to a level close to that of the original RetinaFace. With the introduction of the MSDA attention mechanism, the model’s focus on critical target information was strengthened, and the detection accuracy was further improved.

Compared with RetinaFace, the Precision, Recall, *F*1 Score, and *AP* were improved by 1.78%, 0.22%, 1.01%, and 0.83%, respectively, while the *NME*, FLOPs, Params, and Weight were reduced by 0.02%, 94.71%, 86.70%, and 86.21%, respectively. In summary, the proposed method achieved faster inference speed while maintaining high detection accuracy, demonstrating optimal overall performance, and was capable of meeting the detection requirements and deployment needs in practical sheep farm scenarios.

To further validate the reliability and robustness of the proposed method, five independent experiments were conducted using different random seeds, and the corresponding results are summarized in Table 5. Based on the results of different random-seed experiments, the mean, standard deviation, and 95% confidence interval for each evaluation metric were calculated. As can be observed, the proposed method consistently achieved stable and competitive performance across all evaluation metrics. The Precision, Recall, and *F*1 Score remained at high and stable levels among different groups, with mean values of 94.23%, 96.60%, and 95.39%, respectively. Notably, the small standard deviations indicated that these metrics exhibit limited performance fluctuation under different random initializations. Moreover, an average *AP* of 98.11% was obtained, demonstrating strong overall detection performance. The low standard deviation further confirmed the robustness of the proposed approach. Meanwhile, a low mean *NME* of 2.91% was achieved, reflecting accurate and reliable prediction results. The 95% confidence interval for precision was [92.59%, 95.87%], indicating that the model consistently maintains a high precision above 92.5% across different initialization conditions. The confidence interval for *NME* of [2.66%, 3.16%] further verified the consistent accuracy of key point localization. Overall, the consistently high performance and low variability across all metrics demonstrate that the proposed method is robust and insensitive to random seed variations.

### 4.3. Sheep and Goat Face Detection Results

**Figure 13:**
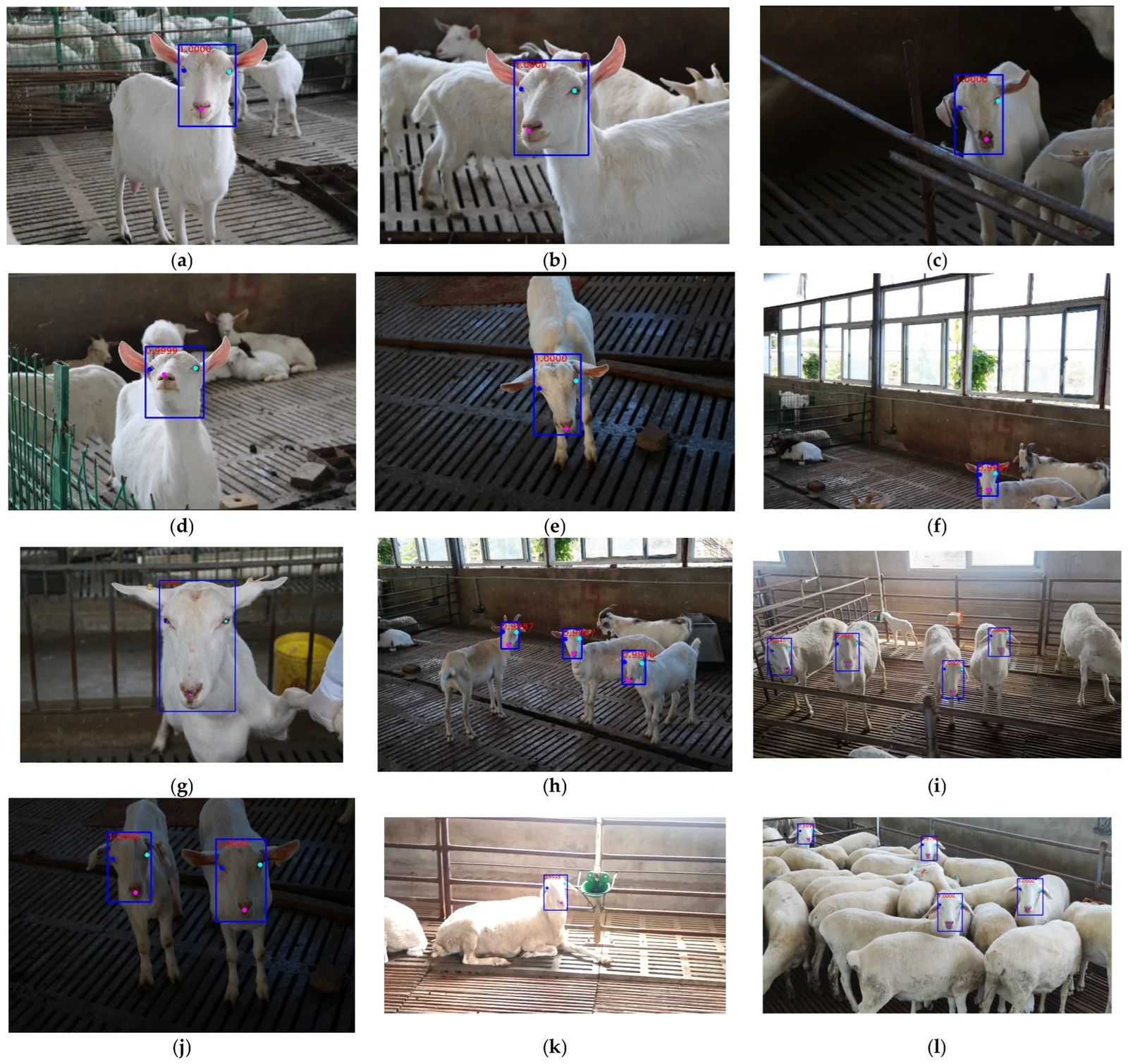
Effect of sheep and goat face detection in different scenes. (**a**) Positive face, (**b**) side face, (**c**) lambs, (**d**) head up, (**e**) head down, (**f**) background interference, (**g**) large scale, (**h**) small scale, (**i**) flock of sheep and goats, (**j**) dim light, (**k**) bright light, and (**l**) stack.

To further validate the model’s detection performance, twelve different scenarios were selected to simulate the living conditions of sheep and goats in an actual farming environment. Sheep and goat face bounding boxes and key point detection were performed, as shown in Figure 13. Figure 13a,b,d,e represent sheep and goat faces from four different angles: positive face, side face, head up and head down. Figure 13g,h depicts large-scale and small-scale sheep and goat faces, while Figure 13j,k illustrates dim and bright-lighting conditions. Figure 13c,f shows environments with lambs and background interference, and Figure 13i,l presents scenes with groups of sheep and goat and stacked sheep and goat. From Figure 13, it can be observed that the proposed model accurately performed bounding box and key point detection for sheep and goat faces across different scenarios, meeting the practical detection needs of sheep and goat farms and providing a foundation for subsequent sheep and goat face recognition.

### 4.4. Jetson Orin Nano Super Device Deployment

**Figure 14:**
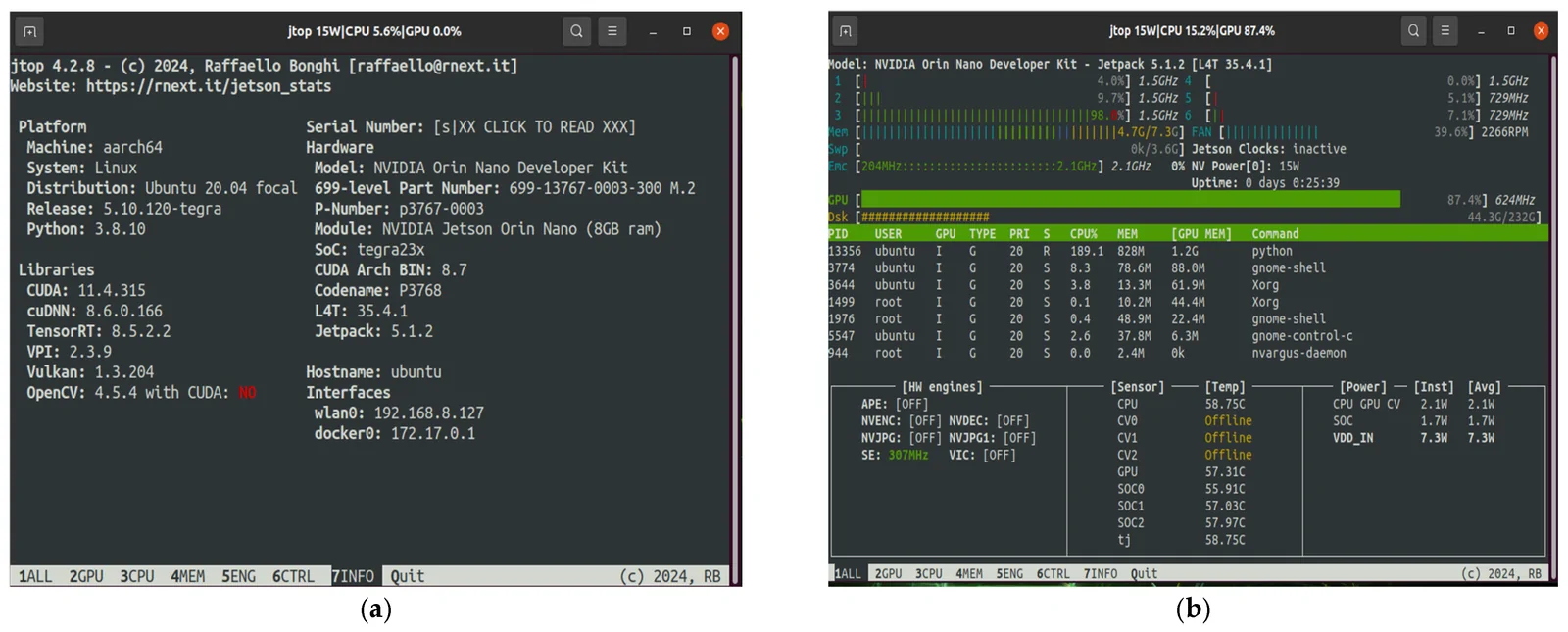
Jetson Orin Nano Super. (**a**) Device software information. (**b**) Device performance consumption.

In this study, the proposed sheep and goat face detection model was integrated with the subsequently developed sheep and goat face recognition model to construct a group sheep and goat identity recognition system, which was deployed on the Jetson Orin Nano Super platform. The software version information of the deployed device is illustrated in Figure 14a. After the deployment was completed, 250 sheep and goat images were used for detection and recognition to evaluate the system performance on the Jetson Orin Nano Super device. During the detection process, the device resource utilization and related performance metrics were randomly captured, as shown in Figure 14b. The experimental results demonstrated that the system achieved an iteration speed of 3.62 it/s with a total detection time of 69 s, corresponding to an average detection time of 276 ms per image.

## 5. Discussion

### 5.1. Feature Analysis of Sheep and Goat Face Detection Network

The lightweight detection scheme suitable for livestock scenes was constructed by introducing variable kernel convolution AKConv and multi-expansion rate MSDA attention mechanism. The loss function was optimized to retain effective key points such as eyes and nose, which accurately obtained the physiological characteristics of sheep and goat faces without clear mouth corners, effectively solving the problem that fixed sampling windows were difficult to adapt to different sizes of sheep and goat faces and complex background interference in feature extraction. In addition, the dataset contained images of different angles, different distances, different light intensities, occlusions, lambs, single sheep and goats and flocks to ensure data diversity. After deduplication and enhancement, 17,436 labeled samples were formed. Compared with the existing public sheep and goat face dataset, the sheep and goat face dataset constructed in this paper had significant advantages in quality, which greatly increased the accuracy of sheep and goat face recognition and provided key technical support for accurate feeding and disease traceability in intelligent animal husbandry.

### 5.2. Model Robustness Analysis

To comprehensively evaluate the robustness of the proposed sheep face detection method in real farming environments, the model robustness was systematically analyzed under various conditions, including different illumination levels, scales, poses, occlusions, and background interferences. A multi-scale dilation attention (MSDA) mechanism was introduced into the SRM module, enabling the model to capture multi-scale contextual information by setting different dilation rates. This approach was suitable for sheep and goat face detection under non-uniform illumination conditions. Figure 13j,k demonstrates that the model can accurately localize sheep and goat faces in both extremely dark and extremely bright environments. The A_EFPN feature fusion network incorporated AKConv into Efficient RepGFPN, allowing simultaneous processing of high-level semantic information and low-level spatial information while maintaining consistent feature depth across scales and facilitating multi-scale information interaction. This design was applicable to multi-scale sheep and goat face detection, and Figure 13g,h shows that the model achieved precise detection of sheep and goat faces across different scale ranges. A multi-task learning strategy was adopted to simultaneously optimize bounding box regression and key point localization, enabling the model to exploit the geometric constraint relationships among three facial key points (left eye, right eye, and nose). Figure 13a–e indicates that the model exhibits satisfactory detection performance under frontal, lateral, head-up, and head-down poses. The SRM module integrated RepNCSPELAN4 and the MSDA attention mechanism to enhance inter-channel information interaction, while the Channel Shuffle operation prevented redundancy and interdependence among feature channels, allowing the model to distinguish sheep faces from visually similar body texture regions. Figure 13c,f demonstrates that the attention mechanism directs the model’s focus toward discriminative facial features under cluttered background conditions.

### 5.3. Model Generalization Analysis

The RetinaFace framework has been successfully applied to human face detection, as summarized in Table 1, and extended to pig face and cattle face recognition. The GhostNet backbone, the MSDA attention mechanism, and the A_EFPN feature fusion network represent architectural-level innovations that were task-agnostic in nature, thereby possessing the potential to be transferred to other animal facial detection tasks. Furthermore, the successful deployment on the Jetson Orin Nano Super platform validated the practical applicability of the proposed lightweight model. The model size of 14.75 MB and the computational cost of 2.36 G FLOPs made it well suited for edge computing devices. Nevertheless, the current deployment evaluation was conducted using only a relatively small test set (250 images), and long-term operational stability was not comprehensively assessed, such as performance degradation during prolonged continuous operation or adaptability to varying hardware conditions.

### 5.4. The Limitations and Prospects of This Study

**Figure 15:**
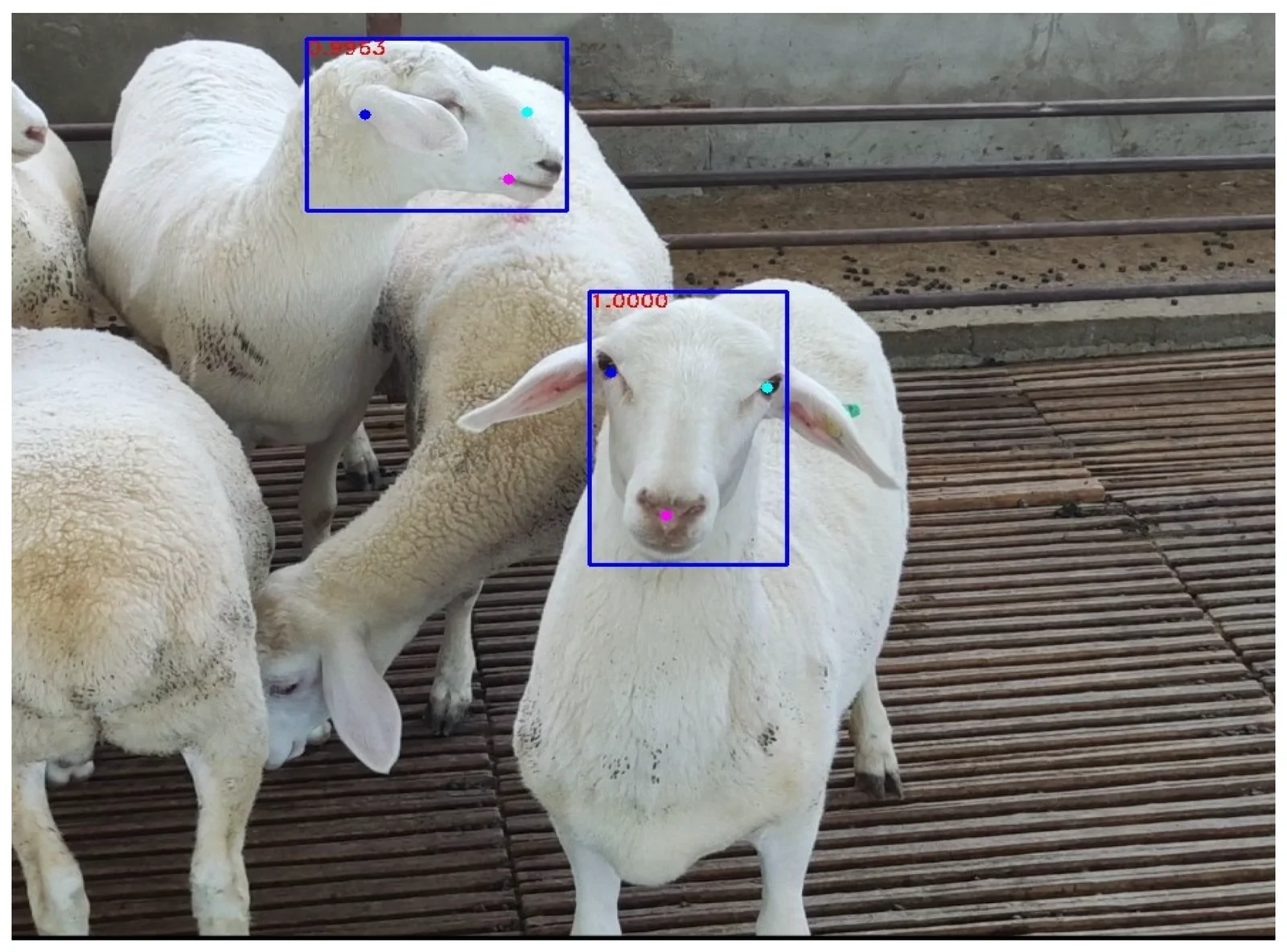
Serious lateral facial key point deviation phenomenon.

The primary emphasis was placed on frontal sheep and goat faces during the sheep and goat face detection process, whereas insufficient consideration was given to cases in which the sheep and goat face was completely tilted laterally, resulting in the occlusion of facial key points and consequently causing deviations in key point detection, as illustrated in Figure 15. In such images, facial key points and discriminative features were incomplete, thereby adversely affecting subsequent feature extraction and identity recognition; accordingly, these images were excluded from annotation during the data labeling process. A similar phenomenon was also observed in sheep and goats with severe facial occlusion. In future work, sheep and goat face alignment and sheep and goat face recognition methods should be further investigated, and all models should be integrated to establish a unified sheep and goat identity recognition system.

This study primarily relied on visual detection examples from different scenarios in Figure 13 to qualitatively analyze model robustness, without providing quantitative evaluation results stratified by conditions such as illumination intensity, target scale, and occlusion degree. This limitation mainly arose from the fact that fine-grained annotations of the aforementioned environmental attributes were not performed for each image during the dataset construction phase, which prevented systematic condition-stratified performance analysis from being conducted at the current stage. Consequently, the performance boundaries of the model under conditions such as extreme illumination, severe occlusion, and extremely long distance remained unclear, constituting an objective limitation in the experimental design and argumentation completeness of this study. To address this issue, supplementary annotations should be added to the dataset in future sheep-face detection and recognition research, with attribute labels such as illumination levels, occlusion ratios, and scale ranges being annotated for each image. Based on these annotations, test subsets should be divided to systematically evaluate the model’s performance in terms of Precision, Recall, AP, and key point localization error under various conditions, and the failure modes of the model should be further analyzed in depth. By introducing a quantitative and stratified evaluation framework, the robustness of the model could be more comprehensively and objectively verified, thereby providing a more solid quantitative basis for further optimization of the model structure in future work.

## 6. Conclusions

In this paper, based on the RetinaFace framework, GhostNet was used as the feature extraction backbone network to realize the model lightweighting. The SRM module was designed as the primary feature extraction module to enhance the model’s feature extraction capabilities. The A_EFPN feature fusion network was employed to replace FPN, improving the model’s multi-scale detection capabilities. An optimized multi-task joint loss function was developed, retaining three key facial points: the left eye, right eye, and nose, for bounding box and key point detection of sheep and goat faces. The proposed sheep and goat face detection method, with AP, NME, and Weight of 98.31%, 2.40%, and 14.75 MB, respectively, realized the fast and accurate detection of sheep and goat faces. To further verify the detection performance of the model, sheep and goat faces in 12 different scenes were detected, which proved that the model could be applied to the actual farm environment, met the demand for sheep and goat face detection, and provided the basis for subsequent sheep and goat face recognition.

## Figures and Tables

**Table 1:** References related to pig faces and cow faces.

Author (Year)	Object	Methodology	Accuracy (%)	References
Xu et al. (2022)	Pig	Design of Trapezoidal Normalized Pixel Difference (T-NPD) feature based on pig face shape, using Trimmed Mean Attention Mechanism (TMAM) and ResNet50 backbone network.	95.06	[7]
Wang et al. (2023)	Pig	Combination of NAM attention mechanism and ResNet model to design ResNAM network for pig face image feature extraction.	95.28	[8]
Xu et al. (2022)	Cow	Combination of lightweight RetinaFace-mobilenet with ArcFace to propose CattleFaceNet for cow face recognition.	91.30	[9]
Kawagoe et al. (2023)	Cow	Use of a YOLO detector to extract the cow head region and crop face images, followed by transfer learning for recognition.	82.90	[10]
Bergman et al. (2024)	Cow	A video collection system was designed 4 m above the feeding area in a dairy farm, using the YOLOv5 algorithm for cow face detection.	97.80	[11]

**Table 2:** GhostNet network parameters.

Input	Operator	# Exp	# Out	SE	Stride	Blocks
640^2^ × 3	Conv2d	-	16	-	2	1
320^2^ × 16	G-bneck	16	16	-	1	
320^2^ × 16	G-bneck	48	24	-	2	
160^2^ × 24	G-bneck	72	32	-	1	
160^2^ × 32	G-bneck	72	32	1	2	
80^2^ × 32	G-bneck	120	64	1	1	
80^2^ × 64	G-bneck	240	64	-	2	2
40^2^ × 64	G-bneck	200	64	-	1	
40^2^ × 64	G-bneck	184	128	-	1	
40^2^ × 128	G-bneck	184	128	-	1	
40^2^ × 128	G-bneck	480	128	1	1	
40^2^ × 128	G-bneck	672	128	1	1	
40^2^ × 128	G-bneck	672	128	1	2	3
20^2^ × 128	G-bneck	960	256	-	1	
20^2^ × 256	G-bneck	960	256	1	1	
20^2^ × 256	G-bneck	960	256	-	1	
20^2^ × 256	G-bneck	960	256	1	1	

Note: # denotes number; Exp denotes expansion; Out denotes output.

**Table 3:** Performance comparison of different backbone networks.

Backbone	Precision/%	Recall/%	F1/%	AP/%	NME/%	FLOPs/G	Params/M	Weight/MB
ResNet50	94.37	96.84	95.59	97.48	2.42	44.64	27.29	106.98
MobileNetv1	83.85	84.83	84.33	88.39	4.41	1.03	0.43	1.78
MobileNetv3	86.58	93.41	89.86	91.62	3.51	2.88	3.16	12.60
GhostNet	87.51	93.80	90.55	92.38	3.48	2.12	3.11	12.48

**Table 4:** Comparison of the performance of different improved networks.

Method	Precision/%	Recall/%	F1/%	AP/%	NME/%	FLOPs/G	Params/M	Weight/MB
RetinaFace	94.37	96.84	95.59	97.48	2.42	44.64	27.29	106.98
RetinaFace + ➀	87.51	93.80	90.55	92.38	3.48	2.12	3.11	12.48
RetinaFace + ➀ + ➁	87.51	93.80	90.55	92.38	3.48	2.12	3.11	12.48
RetinaFace + ➀ + ➁ + ➂	93.63	97.00	95.29	97.33	2.64	2.35	3.61	14.67
RetinaFace + ➀ + ➁ + ➂ + ➃	96.15	97.06	96.60	98.31	2.40	2.36	3.63	14.75

Note: ➀ Denotes the use of the GhostNet backbone network; ➁ denotes the use of the AKConv module; ➂ denotes the use of the A_EFPN feature fusion network, which consists of five SRM modules; and ➃ denotes the introduction of the MSDA attention mechanism into the SRM module.

**Table 5:** Results of different group experiments.

Seed	Precision/%	Recall/%	*F*1/%	*AP*/%	*NME*/%
42	94.67	96.71	95.68	98.29	2.77
67	93.96	96.57	95.24	98.17	3.06
123	95.38	96.58	95.98	98.15	2.83
3407	92.07	96.45	94.21	97.81	3.18
114,514	95.08	96.67	95.86	98.14	2.72
Mean	94.23	96.60	95.39	98.11	2.91
Standard deviation	1.32	0.10	0.72	0.18	0.20
95% confidence interval	[92.59, 95.87]	[96.48, 96.72]	[94.50, 96.28]	[97.89, 98.33]	[2.66, 3.16]

## Data Availability

The data that support the findings of this study are available from the corresponding author upon reasonable request.

## References

[1] Zhang Y.L., Qiao H.B. (2023). Development modes of animal husbandry and their future trends. Sci. Technol. Wind.

[2] Huang X., Huang F., Hu J., Zheng H., Liu M., Dou Z., Jiang Q. (2026). Automatic Face Detection of Farm Images Based on an Enhanced Lightweight Deep Learning Model. Int. J. Pattern Recognit. Artif. Intell..

[3] Peng Y.X., Yang Z.B., Yan K.Y., Wang H., Zhao L.S. (2023). From artificial to intelligent: Research progress in individual identification technology for cattle. China Anim. Husb. Vet. Med..

[4] Yao Z., Tan H., Tian F., Zhou Y., Zhang C. (2021). Research progress of computer vision technology in intelligent sheep farms. China Feed.

[5] Lu J., Wang W., Zhao K., Wang H. (2022). Recognition and segmentation of individual pigs based on Swin Transformer. Anim. Genet..

[6] Li X., Du J., Yang J., Li S. (2022). When mobilenetv2 meets transformer: A balanced sheep face recognition model. Agriculture.

[7] Xu S., He Q., Tao S., Chen H., Chai Y., Zheng W. (2022). Pig face recognition based on trapezoid normalized pixel difference feature and trimmed mean attention mechanism. IEEE Trans. Instrum. Meas..

[8] Wang R., Gao R., Li Q., Dong J. (2023). Pig face recognition based on metric learning by combining a residual network and attention mechanism. Agriculture.

[9] Xu B., Wang W., Guo L., Chen G., Li Y., Cao Z., Wu S. (2022). CattleFaceNet: A cattle face identification approach based on RetinaFace and ArcFace loss. Comput. Electron. Agric..

[10] Kawagoe Y., Kobayashi I., Zin T.T. (2023). Facial region analysis for individual identification of cows and feeding time estimation. Agriculture.

[11] Bergman N., Yitzhaky Y., Halachmi I. (2024). Biometric identification of dairy cows via real-time facial recognition. Animal.

[12] Xue H., Qin J., Quan C., Ren W., Gao T., Zhao J. (2021). Open set sheep face recognition based on Euclidean space metric. Math. Probl. Eng..

[13] Billah M., Wang X., Yu J., Jiang Y. (2022). Real-time goat face recognition using convolutional neural network. Comput. Electron. Agric..

[14] Zhang H.M., Zhou L.X., Li Y.H., Hao J.Y., Sun Y., Li S.Q. (2022). Sheep face recognition method based on improved MobileFaceNet. J. Agric. Mach..

[15] Ning J.F., Lin J.Y., Yang S.Q., Wang Y.S., Lan X.Y. (2023). Recognition method for dairy goat faces based on improved YOLOv5s. J. Agric. Mach..

[16] Zhang X., Xuan C., Ma Y., Su H. (2023). A high-precision facial recognition method for small-tailed Han sheep based on an optimised Vision Transformer. Animal.

[17] Yu F., Ting L., Yang Y. (2026). Color-adaptive sheep face recognition method based on retinex-guided gradient perception convolution. Agriculture.

[18] Zhang F., Zhao X., Wang S., Qiu Y., Fu S., Zhang Y. (2025). Research on herd sheep facial recognition based on multi-dimensional feature information fusion technology in complex environment. Front. Vet. Sci..

[19] Liu L., Gong Y., Zhao G.Q. (2023). Research progress on 3D face recognition. J. Comput. Eng. Appl..

[20] Zhang Q.H., Zhang Y., Zhang M.Y. (2023). A review of progress in occluded face recognition. Comput. Appl. Res..

[21] Zhong J., Chen T., Yi L. (2023). Face expression recognition based on NGO-BILSTM model. Front. Neurorobot..

[22] Mu Z., Feng L., Shang Y., Liu Q., Hu L., Zhou F., Fu X. (2023). Algorithm Analysis of Face Recognition Robot Based on Deep Learning. Int. J. Pattern Recognit. Artif. Intell..

[23] Deng J., Guo J., Ververas E., Kotsia I., Zafeiriou S. (2020). Retinaface: Single-shot multi-level face localisation in the wild. Proceedings of the IEEE/CVF Conference on Computer Vision and Pattern Recognition (CVPR).

[24] Jiao J., Tang Y.M., Lin K.Y., Gao Y., Ma A.J., Wang Y., Zheng W.S. (2023). Dilateformer: Multi-scale dilated transformer for visual recognition. IEEE Trans. Multimed..

[25] Zhang X., Song Y., Song T., Yang D., Ye Y., Zhou J., Zhang L. (2023). AKConv: Convolutional kernel with arbitrary sampled shapes and arbitrary number of parameters. arXiv.

